# Association and dose–response relationship of plasma magnesium with metabolic syndrome in Chinese adults older than 45 years

**DOI:** 10.3389/fnut.2024.1346825

**Published:** 2024-02-14

**Authors:** Jingxin Yang, Yang Cao, Huidi Zhang, Yichun Hu, Jiaxi Lu, Rui Wang, Jie Feng, Lichen Yang

**Affiliations:** Key Laboratory of Trace Element Nutrition, National Health Commission of the People’s Republic of China, Chinese Center for Disease Control and Prevention, National Institute for Nutrition and Health, Beijing, China

**Keywords:** magnesium, metabolic syndrome, IFG, hypertension, Chinese adults

## Abstract

**Purpose:**

Magnesium (Mg) is an essential nutrient for the maintenance of vital physiological functions. Magnesium deficiency is associated with diseases such as obesity, type 2 diabetes mellitus (T2DM), and metabolic syndrome (MetS); however, conclusions have been inconsistent, and there is a particular lack of evidence regarding this association in Chinese population older than 45 years. This study aimed to assess the association between plasma magnesium and the risk of MetS and its components, the dose–response relationship, and the threshold effect relationship in a Chinese population involving older than 45 years.

**Methods:**

A total of 2,101 individuals were randomly selected from the China Nutrition and Health Surveillance (CNHS) (2015–2017) by considering monitoring points. We used the joint statement of the International Diabetes Federation (IDF) in 2009 to define participants with MetS. The plasma magnesium was tested by inductively coupled plasma mass spectrometry (ICP-MS). The logistic regression and restricted cubic spline (RCS) models were used to analyze the association and dose–response relationship between plasma Mg and MetS and its components.

**Results:**

Compared with the lowest quintile (Q1) for plasma Mg, the odds ratios (ORs) and 95% confidence intervals (95% CI) for MetS, impaired fasting glucose (IFG), hypertension, and triglyceride (TG) elevation at the highest quintile (Q5) were 0.419 (0.301, 0.583), 0.303 (0.221, 0.415), 0.446 (0.322, 0.618), and 0.526 (0.384, 0.720), respectively, with all *p* < 0.05. However, in the components of decreased high-density lipoprotein cholesterol (HDL-C) and central obesity, no trend toward lowering with higher plasma magnesium was observed (*p* = 0.717, *p* = 0.865). These associations were not altered by further adjustment for potential confounding variables, including age, gender, education, nationality, area, residence, body mass index (BMI), and heart rate. The RCS analysis showed that, when plasma magnesium was lower than 0.85 mmol/L, the curve was leveled off, and then, the curve showed a decreasing trend with the increase in plasma magnesium.

**Conclusion:**

Therefore, plasma Mg was negatively associated with MetS and its components (including IFG, hypertension, and elevated TG) in people older than 45 years. In addition, plasma Mg greater than or equal to 0.85 mmol/L, which is higher than the commonly used threshold of 0.75 mmol/L, may be protective against MetS and its components (including elevated FPG, elevated blood pressure, and elevated TG). More prospective studies, such as randomized controlled trials, are necessary to confirm the effective impact of Mg on MetS and its components. Plasma Mg levels in the MetS population older than 45 years require attention.

## 1 Introduction

Magnesium (Mg) is one of the essential nutrients for maintaining important physiological functions of the body. It is involved in many fundamental processes, and Mg deficiency is often associated with negative health outcomes (1). Mg is involved in more than 300 enzymatic reactions and significantly influences neurotransmitter release, oxidative stress prevention, bone metabolism, heart rhythm, and vascular tone (2). Mg deficiency is frequent in obese patients, individuals with type 2 diabetes (T2DM), and those with metabolic syndrome (MetS). The symptoms of Mg deficiency are usually non-specific and may be mistaken for an inadequate intake of other nutrients. Typically, according to the Nutrition and Health Status Monitoring Report of Chinese Residents 2010–2013 (3), the prevalence of MetS has increased significantly from the age of 45 years. Although the relationship between plasma Mg and the body Mg content may not be obvious, plasma Mg remains the most widely used indicator of Mg nutrition status, and there is still a lack of reports on Mg nutrition status in Chinese people older than 45 years (4).

The MetS is characterized by a clustering of cardiovascular risk factors, including central obesity, elevated blood pressure, dyslipidemia (high triglycerides), low high-density lipoprotein cholesterol (HDL-C), and fasting hyperglycemia (5). MetS is on the rise globally and is clinically important due to its association with cardiovascular disease, T2DM, and cancer (6). Although the pathogenesis of MetS is not well defined, Mg may play a role in the development of MetS, insulin resistance, and chronic low-grade inflammation due to central obesity, which are the most widely accepted underlying reasons (7). Lifestyle risk factors such as obesity, physical inactivity, smoking, and unhealthy diet are strongly associated with the risk of developing MetS (8). Nikniaz et al. (9) study also demonstrated that a multi-mineral-based dietary pattern including Mg is associated with healthier metabolic factors in the Iranian population. However, studies exploring the relationship between plasma Mg and MetS and its components as well as whether there is a dose-effect or threshold-effect relationship in Chinese population have been relatively limited.

Guerrero-Romero et al. (10) review of clinical evidence from randomized controlled trials assesses the efficacy of Mg supplementation in improving the composition of MetS. Their results suggest that supplementation for people with hypomagnesemia can effectively treat MetS. Champagne (11) review also recognized that adequate Mg intake is beneficial in controlling blood pressure, promoting weight loss, and improving the risk of chronic diseases. While data are not entirely consistent, Sarrafzadegan et al. systematic review and meta-analysis (12) found a negative correlation between Mg intake and MetS. However, this negative correlation is highly heterogeneous and sensitive.

As mentioned above, the nutritional status of Mg may be associated with MetS. Moreover, the surveillance results in China have shown that the prevalence of MetS has begun to increase in the Chinese population older than 45 years, which is a cause for concern. Studies on MetS and plasma Mg, especially in the Chinese population older than 45 years, were scarce. Therefore, we aimed to explore the relationship between plasma Mg and MetS in Chinese adults older than 45 years, especially the dose–response relationship and threshold-effect relationship.

## 2 Materials and methods

### 2.1 Study population

The nationally representative China Nutrition and Health Monitoring (2015) (CNHS 2015) was the data source for this study. Protocols for monitoring the sample selection have been published elsewhere (13). According to the design of natural population distribution, a representative sample set of people older than 45 years was selected from approximately 180,000 monitoring populations by the stratified sampling method. The formula for calculating the sample size of this cross-sectional study is $N=deff\frac{u^{2}p\left(1-p\right)}{d^{2}}$ . According to Wang et al. result, in China, in 2013 the prevalence of diabetes in 2013 was 10.9% (14). The values of the parameters *u*, *p*, *deff*, and *d* were 1.96, 0.109, 1.5, and 4%, respectively. The calculation yields a minimum sample size of 350 for each stratification factor. Taking into account gender and geographic location (east, mid, and west), there were six strata, and the whole cluster was randomly sampled with a population of 2,101 participants based on the distribution of 302 monitoring sites in China. Detailed sampling strategies are available in the previous article. All subjects in this study signed informed consent before the start of the trial. This study is also in line with the Declaration of Helsinki. This study has also been approved by the ethics committee of the Institute of Nutrition and Health, Chinese Center for Disease Control and Prevention, Changping district, Beijing, China (No. 201519-A).

### 2.2 Criteria for inclusion and diagnosis of MetS

The criteria we use for MetS, according to the joint statement of the International Diabetes Federation (IDF) in 2009 (15), recognize MetS when any three of the following conditions are present: (1) Elevated plasma glucose: Diagnosed with diabetes, taking hypoglycemic medications, or having fasting blood glucose (FPG) ≥ 5.6 mmol/L. (2) Elevated blood pressure (BP): systolic blood pressure (SBP) ≥130 mmHg, diastolic blood pressure (DBP) ≥85 mmHg, or under treatment with medications for essential hypertension. (3) Central obesity: waist circumference (WC) ≥ 85 cm for men and 80 cm for women. (4) Triglyceride (TG) elevation: ≥ 1.7 mmol/L or receiving treatment. (5) Reduced high-density lipoprotein cholesterol (HDL-C): < 1.0 mmol/L for men and < 1.3 mmol/L for women or receiving relevant medication.

### 2.3 Basic information and laboratory measurements

Each participant was asked to fast for more than 8 h, and 5 mL of venous blood was collected and centrifuged at 3000 × g for 15 min in separate tubes. Separated plasma was stored in a refrigerator at 80°C pending relevant testing. Training professionals used a nationally standardized questionnaire to collect basic information such as name, gender, ethnicity, education level, smoking, and alcohol consumption. Height, weight, WC, and BP were measured by a trained professional three times for each individual, and the measurements were averaged. Body mass index (BMI) was calculated by dividing weight (kg) by the square of height (m^2^). Non-smokers and ex-smokers who have quit smoking were judged to be non-smokers, and non-drinkers and ex-drinkers who have quit drinking were judged to be non-drinkers. The residence was categorized as urban and rural. Geographic location was divided into east, central, and west according to longitude. Educational attainment was divided into elementary school and below, middle school and high school, and college and above.

The study used an automatic biochemical analyzer (Hitachi 7600, Tokyo, Japan) to measure the liver and renal function, including fasting plasma glucose (FPG), plasma lipids, including total cholesterol (TC), triglyceride (TG), high-density lipoprotein cholesterol (HDL-C), low-density lipoprotein cholesterol (LDL-C), and plasma uric acid (UA). Glycosylated hemoglobin (HbA1c) was determined by a high-performance lipid chromatography (HPLC) method using Trinity Biotech Premier Hb9210 (Dublin, Ireland). In this study, plasma Mg was determined by inductively coupled plasma mass spectrometry (ICP-MS, PerkinElmer, NexION 350, Waltham, MA, United States). Plasma Mg was measured with 0.5% (v/v) high-purity nitric acid diluted with plasma in a 1:20 ratio. Standard quality control (QC) assays were performed on every 20 samples. QC consisted of Seronorm (Level 2, Billingstad, Norway) and ClinChek (Level 2, Munich, Germany). The coefficient of variation for Mg was 3.56% between batches and 2.30% within batches.

### 2.4 Statistical analysis

Quantitative information was expressed in the form of mean ± SD, and qualitative information was expressed in the form of percentage. An analysis of variance (ANOVA) and the chi-squared tests were used to compare the demographic characteristics of individuals with different plasma Mg levels. In this study, multiple logistic regression was applied to explore the relationship between plasma Mg and MetS and its components. Different variables were added to the model to compare the odds ratios (ORs) and 95% confidence intervals (95% CI) with the results of the reference quartile (lowest quartile, Q1) under different components in the model. We also used restricted cubic spline (RCS) to test for non-linear relationships and to explore the shape of the dose–response relationship between plasma Mg and MetS and their different components. The statistical analysis was conducted using SAS version 9.4 software (SAS Institute Inc., Cary, NC, United States). All *p*-values were two-sided, and the differences were considered statistically significant with the *p*-values less than or equal to 0.05.

## 3 Results

### 3.1 Basic characteristics of 2,101 participants

The mean plasma Mg was 0.88 ± 0.10 mmol/L. Based on the plasma Mg levels, 2,101 subjects were equally divided into five parts to compare the various influences on different plasma Mg levels in Table 1. As plasma Mg levels increased, the percentage of obese, WC, TC, TG, LDL-C, FPG, HbA1c, systolic blood pressure (SBP), diastolic blood pressure (DBP), and heart rate showed a tendency to decrease, and the difference was statistically significant (*p* < 0.05). Other variables including age, gender, nationality, education, region, residence, smoking or drinking status, HDL-C, and UA did not show statistically significant differences between the different plasma Mg groups (*p* > 0.05).

**Table 1:** Characteristics of 2,101 participants according to the quintiles of plasma Mg.

Variables	Quintile of plasma Mg (mmol/L)					*p*-value
	Q1(≤0.805)	Q2(≤0.852)	Q3(≤0.895)	Q4(≤0.950)	Q5(>0.950)	
N	419	419	420	424	419	
Gender						0.825
Man	51.10%	49.40%	51.00%	47.40%	50.10%	
Woman	48.90%	50.60%	49.00%	52.60%	49.90%	
Nationality						0.481
Han	86.60%	88.50%	89.00%	89.60%	90.50%	
Ethnic minorities	13.40%	11.50%	11.00%	10.40%	9.50%	
Age group						0.361
45<age ≤ 55	23.20%	29.10%	24.00%	25.90%	28.20%	
55<age ≤ 65	36.30%	32.90%	35.50%	29.00%	33.20%	
65<age ≤ 75	19.60%	19.60%	18.60%	22.40%	17.70%	
age>75	21.00%	18.40%	21.90%	22.60%	21.00%	
BMI group						0.033
<18.5	4.30%	2.90%	3.10%	5.20%	5.00%	
18.5–23.9	44.40%	45.80%	47.10%	46.00%	54.90%	
24–27.9	33.90%	34.40%	35.20%	35.60%	29.10%	
≥28	17.40%	16.90%	14.50%	13.20%	11.00%	
Education						0.088
Elementary school and blow	65.40%	57.80%	57.90%	56.60%	59.70%	
Middle school and high school	33.70%	39.60%	40.50%	41.70%	39.60%	
College and above	1.00%	2.60%	1.70%	1.70%	0.70%	
Area						0.252
East	31.50%	33.90%	36.00%	38.70%	37.00%	
Mid	32.20%	28.40%	27.10%	30.20%	30.80%	
West	36.30%	37.70%	36.90%	31.10%	32.20%	
Residence						0.357
City	60.40%	57.30%	57.10%	60.80%	63.00%	
Rural area	39.60%	42.70%	42.90%	39.20%	37.00%	
Smoke						0.158
Yes	29.60%	23.60%	26.70%	22.90%	24.30%	
No	70.40%	76.40%	73.30%	77.10%	75.70%	
Drink						0.949
Yes	32.90%	32.50%	34.00%	31.60%	33.70%	
No	67.10%	67.50%	66.00%	68.40%	66.30%	
BMI	24.25 ± 3.75	24.46 ± 3.56	24.30 ± 3.60	23.97 ± 3.55	23.60 ± 3.33	0.004
Waist (cm)	83.84 ± 11.25	84.11 ± 10.51	83.78 ± 11.26	82.78 ± 10.59	82.19 ± 10.46	0.048
TC (mmol/L)	4.96 ± 1.08	4.81 ± 0.98	4.79 ± 0.99	4.93 ± 1.01	4.69 ± 0.91	<0.001
TG (mmol/L)	1.66 ± 1.19	1.53 ± 1.26	1.45 ± 0.98	1.60 ± 1.30	1.33 ± 0.87	<0.001
LDL-C (mmol/L)	3.12 ± 0.93	3.01 ± 0.84	2.97 ± 0.87	3.05 ± 0.88	2.88 ± 0.80	0.002
HDL-C (mmol/L)	1.30 ± 0.34	1.27 ± 0.32	1.31 ± 0.33	1.30 ± 0.33	1.32 ± 0.30	0.269
SBP (mmHg)	144.35 ± 23.89	140.29 ± 22.65	141.61 ± 22.61	140.91 ± 21.34	135.61 ± 20.34	<0.001
DBP (mmHg)	81.19 ± 12.41	79.76 ± 10.93	79.67 ± 11.60	79.29 ± 11.02	77.19 ± 10.53	<0.001
FPG (mmol/L)	6.57 ± 2.80	5.84 ± 1.75	5.64 ± 1.30	5.60 ± 1.36	5.22 ± 0.88	<0.001
HbA1c (%)	5.66 ± 1.66	5.25 ± 1.06	5.17 ± 0.87	5.12 ± 0.87	4.95 ± 0.57	<0.001
UA	321.17 ± 96.36	311.98 ± 85.05	316.13 ± 83.41	307.40 ± 78.59	308.01 ± 86.17	0.108
Heart rate	80.11 ± 35.78	77.68 ± 19.42	76.17 ± 19.07	75.37 ± 10.93	76.09 ± 18.18	0.016

BMI, Body mass index; TC, total cholesterol; TG, triglyceride; LDL-C, low-density lipoprotein cholesterol; HDL-C, high-density lipoprotein cholesterol; SBP, systolic blood pressure; DBP, diastolic blood pressure; FPG, fasting plasma glucose; HbA1c, glycosylated hemoglobin; UA, uric acid.

### 3.2 Multivariate logistic regression analysis of plasma Mg and MetS and their components

Table 2 summarizes the results of the multivariate logistic analysis of the relationship between plasma Mg and MetS and its components. In the context of MetS as a whole, there was a gradual and statistically significant decrease in ORs with increasing plasma Mg levels, regardless of correction for other possible confounders. Model 1 was an uncorrected model, model 2 corrected for age, gender, education, nationality, area, and residence, and model 3 further corrected for BMI and heart rate. Its components, including impaired fasting glucose (IFG), hypertension, and elevated TG, also showed a tendency to decrease with elevated plasma Mg levels. Compared with Q1 for plasma Mg, the ORs (95% CI) for MetS, IFG, hypertension, and TG elevation at Q5 were 0.419 (0.301, 0.583), 0.303 (0.221, 0.415), 0.446 (0.322, 0.618), and 0.526 (0.384, 0.720), respectively, with all the *p-values* less than 0.05. However, in the components of decreased HDL and central obesity, no trend toward lowering with higher plasma Mg was observed (*p* = 0.717, *p* = 0.865).

**Table 2:** Multivariate logistic regression analysis of plasma Mg and MetS and its components [OR (95%CI)].

	Quintile of plasma Mg (mmol/L)					*p*-value
	Q1(≤0.805)	Q2(≤0.852)	Q3(≤0.895)	Q4(≤0.950)	Q5(>0.950)	
MetS						
Model 1	1(ref)	0.918 (0.700, 1.203)	0.753 (0.574, 0.989)	0.741 (0.565, 0.973)	0.426 (0.321, 0.566)	<0.001
Model 2	1(ref)	0.909 (0.689, 1.200)	0.732 (0.554, 0.967)	0.682 (0.516, 0.901)	0.396 (0.296, 0.529)	<0.001
Model 3	1(ref)	0.855 (0.622, 1.176)	0.678 (0.491, 0.936)	0.713 (0.517, 0.982)	0.419 (0.301, 0.583)	<0.001
IFG						
Model 1	1(ref)	0.834 (0.636, 1.094)	0.677 (0.515, 0.889)	0.556 (0.422, 0.733)	0.296 (0.220, 0.398)	<0.001
Model 2	1(ref)	0.814 (0.618, 1.072)	0.652 (0.494, 0.861)	0.518 (0.391, 0.686)	0.274 (0.203, 0.371)	<0.001
Model 3	1(ref)	0.807 (0.603, 1.078)	0.675 (0.504, 0.904)	0.564 (0.420, 0.758)	0.303 (0.221, 0.415)	<0.001
Hypertension						
Model 1	1(ref)	0.885 (0.651, 1.203)	0.799 (0.590, 1.083)	0.810 (0.598, 1.097)	0.454 (0.339, 0.609)	<0.001
Model 2	1(ref)	0.950 (0.686, 1.316)	0.796 (0.577, 1.100)	0.773 (0.560, 1.069)	0.428 (0.313, 0.585)	<0.001
Model 3	1(ref)	0.902 (0.644, 1.265)	0.756 (0.541, 1.057)	0.780 (0.558, 1.090)	0.446 (0.322, 0.618)	<0.001
Hyperlipidemia—increased TG						
Model 1	1(ref)	0.709 (0.532, 0.944)	0.798 (0.601, 1.058)	0.486 (0.360, 0.657)	0.612 (0.502, 0.745)	<0.001
Model 2	1(ref)	0.654 (0.488, 0.875)	0.690 (0.516, 0.921)	0.763 (0.573, 1.015)	0.472 (0.348, 0.639)	<0.001
Model 3	1(ref)	0.630 (0.465, 0.852)	0.683 (0.505, 0.923)	0.817 (0.607, 1.100)	0.526 (0.384, 0.720)	0.001
Dyslipidemia—decreased HDL-C						
Model 1	1(ref)	1.021 (0.771, 1.351)	0.996 (0.753, 1.319)	0.942 (0.711, 1.247)	0.758 (0.569, 1.009)	0.238
Model 2	1(ref)	1.005 (0.748, 1.350)	0.994 (0.740, 1.335)	0.878 (0.653, 1.180)	0.722 (0.534, 0.976)	0.149
Model 3	1(ref)	0.992 (0.730, 1.348)	0.992 (0.728, 1.350)	0.932 (0.685, 1.269)	0.823 (0.602, 1.126)	0.717
Central obesity						
Model 1	1(ref)	0.894 (0.681, 1.175)	0.884 (0.674, 1.161)	0.721 (0.550, 0.947)	1.328 (1.094, 1.611)	0.147
Model 2	1(ref)	0.960 (0.726, 1.269)	0.884 (0.669, 1.167)	0.835 (0.633, 1.103)	0.686 (0.519, 0.906)	0.069
Model 3	1(ref)	0.857 (0.584, 1.258)	0.819 (0.558, 1.201)	0.937 (0.640, 1.373)	0.881 (0.605, 1.283)	0.865

IFG, impaired fasting glucose; model 1 unadjusted; model 2 further added age, gender, education, nationality, area, and residence; model 3 further added BMI and heart rate.

### 3.3 Dose–response relationship between plasma Mg and MetS and its components

**Figure 1:**
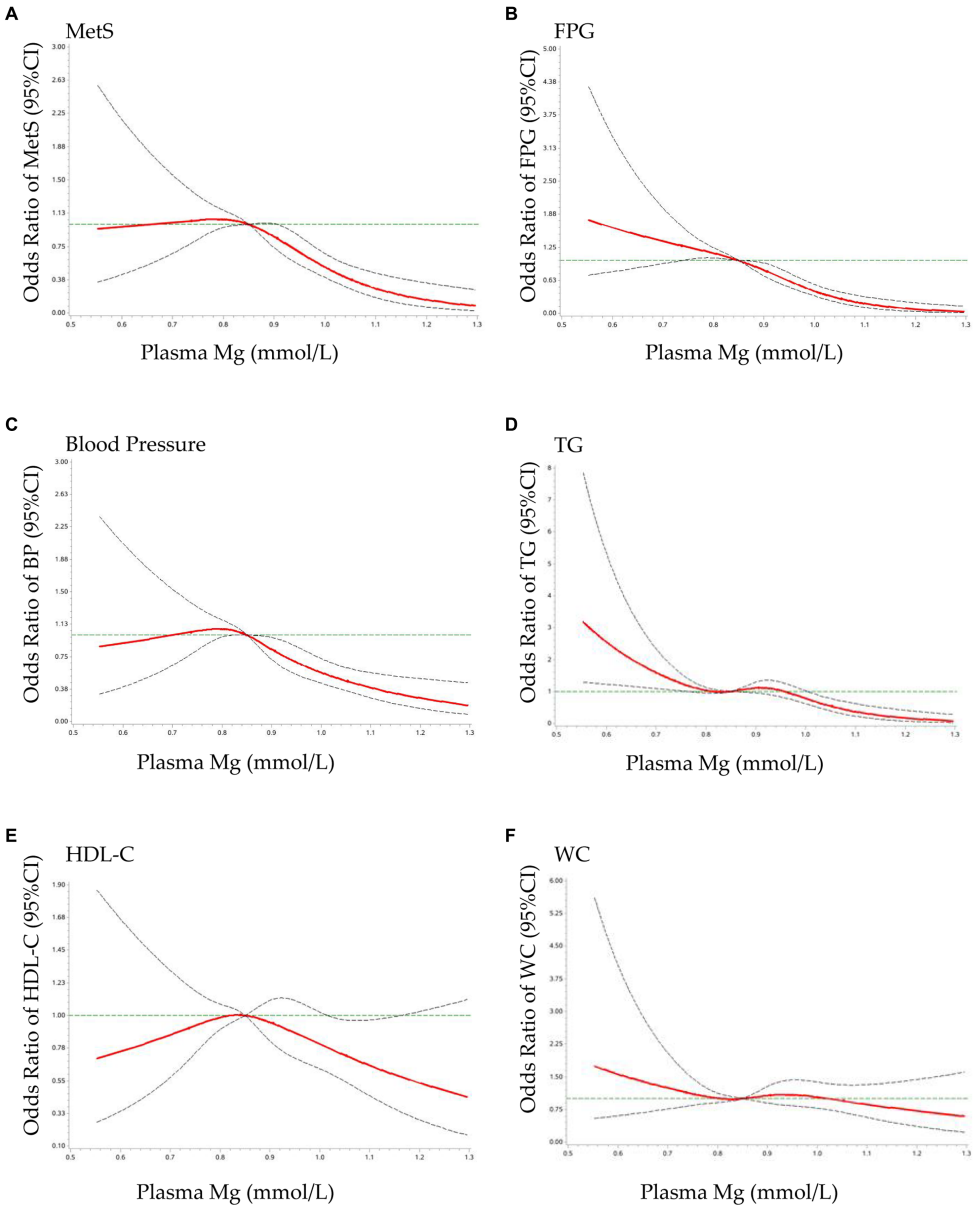
Dose–response relationship of plasma Mg with MetS and its components OR: The ORs (red solid lines) and 95% CIs (dashed lines) of plasma Mg are indicated by straight lines based on restrictive cubic splines (RCS). Gender, area, age, education, residence, SBP, DBP, BMI, WC, TG, LDL-C, and UA were adjusted. **(A)** MetS, metabolic syndrome; **(B)** FPG, fasting plasma glucose; **(C)** blood pressure; **(D)** TG, triglyceride; **(E)** HDL-C, high-density lipoprotein cholesterol; **(F)** WC, waist circumference.

The results of the RCS analysis are shown in Figure 1. When plasma Mg levels were greater than 0.85 mmol/L, there was a significant protective effect against elevated MetS (a), FPG (b), increased blood pressure (c), and elevated TG (d), with no significant effect on the reduction of HDL-C (e), or the increase in waist circumference (f). MetS showed a flattening and then a decreasing trend with increasing plasma Mg, with a cutoff point of approximately 0.85 mmol/L, which is similar to the relationship between blood pressure and plasma Mg. FPG tended to decrease as plasma Mg increased and was not statistically significant until 0.85 mmol/L. There was a statistically significant tendency for blood glucose to decrease when plasma Mg was greater than 0.85 mmol/L, which is similar to the relationship between TG and plasma Mg.

## 4 Discussion

In this study, we analyzed the association and dose–response relationship between plasma Mg and MetS in adults older than 45 years using the nationally representative data from China. The results showed that, when the plasma Mg concentration was greater than 0.85 mmol/L, the OR values of the increased MetS, FPG, BP, and TG were significantly lower than 1 but had no significant effect on the decrease of HDL and WC.

The present study obtained a significantly negative correlation between overall MetS and plasma Mg levels. Huang et al. (16) recruited 1,277 adults to evaluate the relationship between metal mixture exposure and the prevalence of MetS in Chinese middle-aged and elderly populations. They also used ICP-MS and RCS methods to detect the plasma levels of 13 metals and the dose–response relationships of plasma metals with MetS, respectively. The results showed that the concentrations of Mg were lower in the MetS group (*p* < 0.05), and the adjusted OR (95% CI) in the highest quartile was 0.44 (0.35, 0.76) compared with the lowest quartile. It was demonstrated that Mg and Mo were the major contributors to the combined effect, and elevated plasma Mg levels were associated with a reduced prevalence of MetS. Except for Huang’s cross-sectional research, Afitska et al. RCT study also confirms this. Afitska et al. (17) recruited 50 participants with normal plasma Mg status and randomly assigned them to 400 mg/day Mg citrate or placebo for 12 weeks. Compared with the placebo group, Mg supplementation resulted in a statistically significant reduction in SBP and DBP (145 ± 10 vs. 121 ± 5 mmHg and 85 ± 3 vs. 79 ± 3 mmHg) along with a statistically significant reduction in HbA1c (6.43 ± 0.64% vs. 6.15 ± 0.55%). Thus, this study confirms that oral Mg citrate supplementation even when given to individuals with normal plasma Mg with MetS reduces components of MetS such as HbA1c and increased blood pressure.

The negative correlation between elevated TG and plasma Mg found in this study has consistent results in other studies. Rayssiguier et al. (18) review showed that Mg deficiency leads to stress effects and increased susceptibility to stress-generated physiological damage. Inflammation occurring during experimental Mg deficiency induces hypertriglyceridemia and primary atherosclerosis changes in lipoprotein metabolism. Guerrero-Romero et al. (19) cross-sectional study enrolled 427 men and non-pregnant women aged 20–65 years and also showed that hypomagnesemia is strongly associated with hypertriglyceridemia and insulin resistance in obese individuals.

In addition to the negative correlation with MetS, this study also found a negative correlation between plasma Mg levels and FPG. Del Gobbo et al. (20) included 16 prospective studies comprising 313,041 individuals with 11,995 cardiovascular disease (CVD), 7,534 ischemic heart disease (IHD), and 2,686 fatal IHD events. The results found that each 0.2 mmol/L increase in plasma Mg was associated with a 30% reduction in the risk of CVD (RR: 0.70; 95% CI: 0.56, 0.80), a lower risk of IHD (RR: 0.83; 95% CI: 0.75, 1.05), and lethal IHD (RR: 0.61; 95% CI: 0.37, 1.00). Increasing dietary Mg by 200 mg/day was not significantly associated with cardiovascular disease (RR: 0.89; 95% CI: 0.75, 1.05) but was associated with a 22% lower risk of IHD (RR: 0.78; 95% CI: 0.67, 0.92). Dietary Mg was non-linear (*p* = 0.001) and negatively associated with lethal IHD compared to those with lower intakes, with an observed threshold of 250 mg/day (RR: 0.73; 95% CI: 0.62, 0.86). In conclusion, this meta-analysis study found that circulating and dietary Mg were inversely associated with the risk of cardiovascular disease. Zhang et al. (21) recruited 254 patients with T2DM to determine the relationship between serum Mg and the peripheral nerve function in patients with T2DM. The results showed that serum Mg levels were significantly lower in patients with diabetic peripheral neuropathy (DPN). The percentage of DPN was lower in T2DM patients with higher serum Mg levels, which suggests a correlation between plasma Mg levels and diabetic complications of DPN. Albaker et al. (22) conducted an RCT clinical trial to evaluate the effect of adding Mg chloride supplements to desalinated water consumed by T2DM patients on blood glucose, metabolic parameters, and insulin sensitivity indices. A total of 102 T2DM patients completed the trial, and the results showed that adding a dose (50 mg/L in this study) of Mg to drinking water improves long-term glycemic control indices and reduces insulin resistance in T2DM patients. Das (23) also suggests that Mg promotes the metabolism of essential fatty acids (EFAs), which, in turn, protects pancreatic b-cells, improves insulin sensitivity, and suppresses inflammation, which leads to the protective role of Mg against T2DM.

This study also found a negative correlation between blood pressure (BP) and plasma Mg. Several intervention studies also demonstrated that oral Mg supplementation lowered BP in patients with mild-to-moderate hypertension. Zhang et al. (24) meta-analysis included 34 RCTs and involved 2,028 participants to quantify the effect of oral Mg supplementation on BP. Their meta-analysis showed that a 3-month Mg supplementation of 368 mg/day significantly reduced SBP and DBP by 2.00 mmHg and 1.78 mmHg, respectively. The RCS analysis showed that the addition of 300 mg/day for 1 month was sufficient to raise serum Mg and lower blood pressure. Furthermore, serum Mg was negatively correlated with DBP and not significantly correlated with SBP (*p* < 0.05). Kass et al. (25) meta-analysis included 141 articles, which also certified that Mg supplementation can achieve a small but clinically significant reduction in BP. Witteman et al. (26) recruited 91 middle-aged and elderly women with mild-to-moderate hypertension who were randomized to 6 months of treatment with aspartate-Mg hydrochloride (20 mmol/d) or placebo with the same appearance. The results showed that the SBP and DBP of Mg-supplemented group decreased by 2.7 mmHg and 3.4 mmHg, respectively.

No dose–response relationship between plasma Mg and blood HDL-C and WC was found in this study. However, contrary to our findings, Salehidoost et al. (27) conducted a 12-week RCT, which recruited 86 prediabetic patients who were given Mg oxide 250 mg/day versus placebo, and the results showed that Mg supplementation increased HDL-C levels in patients with prediabetes. However, Mg supplementation did not improve other cardiometabolic indices, such as HOMA-IR index, TC, LDL-C, TG, UA, and C-reactive protein (CRP). Some studies are consistent with our findings, such as Simental-Mendía et al. (28) research. LUIS performed a meta-analysis of 18 RCTs to evaluate the effect of oral Mg supplementation on lipids in diabetic and non-diabetic patients. The results of this meta-analysis suggest that Mg supplementation does not have a significant effect on lipid profiles, including TC (*p* = 0.671), LDL-C (*p* = 0.903), HDL-C (*p* = 0.076), and TG concentrations (*p* = 0.149) in diabetic or non-diabetic individuals.

This study also found that BMI tended to decrease with increasing plasma Mg levels, and the difference was statistically significant. Singh et al. (29) research also found that serum Mg/insulin ratio is negatively associated with high body fat percentage. They randomly selected 850 men aged 25–64 years to determine the relationship between high body fat percentage and serum Mg levels in an urban Indian population. The results showed that Mg deficiency (OR: 1.02) was a risk factor for high body fat rate and central obesity. Hosseini et al. (30) reviewed 31 articles, which also demonstrated a negative correlation between plasma Mg and obesity. At the same time, contrary studies, such as Asbaghi et al. study, are showing that plasma Mg is not associated with obesity, OMID summarized five randomized controlled trials that showed that Mg supplementation did not affect weight (WMD: −0.01 kg), BMI (WMD: −0.07), and WC (WMD: 0.12) (31).

The study also identified 0.85 mmol/L as a critical inflection point for MetS and its three components: elevated FPG, elevated TG, and elevated blood pressure. Other research groups, including those in the US (32) and German (33), proposed that the lower cutoff value of the Mg deficiency should be 0.85 mmol/L. They all agreed that the range of reference values usually found for serum Mg (0.75–0.95 mmol/L), especially the lower reference of 0.75 mmol/L, is no longer universally applicable. This is because subclinical Mg deficiency may still exist despite the normal performance within the current serum Mg reference. According to the current data, serum Mg values below 0.85 mmol/L are associated with increased health risks. Therefore, the lower limit of the reference range should be raised to 0.85 mmol/L. Rosanoff et al. (34) consensus also suggests an updated standardization of serum Mg reference ranges. Andrea argued that it would be more appropriate to standardize the lower reference value for serum Mg to 0.85 mmol/L (2.07 mg/dL; 1.7 mg eq/L) for proper diagnosis, awareness, and management of Mg status. Our study also supports 0.85 mmol/L as a reasonably low value for disease prevention.

## 5 Strengths and limitations

This study has several advantages. First, the sample was a representative sample randomly selected from nationally representative data (CNHS 2015) with a complete quality control system. Second, we used the more recognized ICP-MS method for plasma Mg with comparable experimental results. Third, we synthesized the relationship between plasma Mg and MetS and its components using an RCS approach and obtained a meaningful inflection point value of 0.85 mmol/L.

This study also has some disadvantages that need to be mentioned. First, due to the use of cross-sectional data, causal associations could not be determined. Second, as the data used were nationally monitored and included only some common physiological and biochemical factors, there may be other confounding factors that are not measured and controlled for, causing the possibility of error and bias.

## 6 Conclusion

In conclusion, plasma Mg was negatively associated with MetS and its components (including IFG, hypertension, and elevated TG) in people older than 45 years. In addition, plasma Mg greater than or equal to 0.85 mmol/L, which is higher than the commonly used threshold of 0.75 mmol/L, may be protective against MetS and its components (including elevated FPG, elevated blood pressure, and elevated TG). More prospective studies, such as randomized controlled trials, are necessary to confirm the effective impact of Mg on MetS and its components. The plasma Mg levels in the MetS population older than 45 years require attention.

## Data availability statement

The data set presented in this article is not readily available as it belongs to China Nutrition and Health Surveillance (2015) (CNHS 2015). The database is not publicly available. Requests regarding the datasets should be directed to 243671926@qq.com.

## Ethics statement

The studies involving humans were approved by the National Institute for Nutrition and Health, Chinese Center for Disease Control and Prevention. The studies were conducted in accordance with the local legislation and institutional requirements. Written informed consent for participation in this study was provided by the participants' legal guardians/next of kin.

## Author contributions

JY: Writing – original draft. YC: Investigation, Writing – review & editing. HZ: Methodology, Writing – review & editing. YH: Investigation, Writing – review & editing. JL: Investigation, Writing – review & editing. RW: Investigation, Writing – review & editing. JF: Formal analysis, Writing – review & editing. LY: Funding acquisition, Writing – review & editing.
